# Case Report of Distal Radioulnar Joint and Posterior Elbow Dislocation

**DOI:** 10.21980/J89S6K

**Published:** 2020-10-15

**Authors:** Danielle Matonis, Katelyn Wittel, Alisa Wray

**Affiliations:** * University of California, Irvine, Department of Emergency Medicine, Orange, CA

## Abstract

The distal radioulnar joint plays a critical role in pronation and supination of the forearm and can be injured with traumatic axial loading of the wrist. This is a case report of a 36-year-old male who presented to the emergency department with left upper extremity pain after falling onto an outstretched arm. The physical exam showed swelling and decreased range of motion of the elbow and wrist due to pain. Radiographs demonstrated left posterolateral elbow dislocation and left dorsal distal radioulnar joint dislocation. The patient underwent a successful closed reduction and splinting of both the left elbow and left distal radioulnar joint with good outcomes on follow up a week later. Since elbow dislocations are usually easily recognized and commonly treated in the emergency department, this case report focuses on the rarer finding of associated distal radioulnar joint dislocation because these injuries are often underdiagnosed or missed on initial assessment.

**Topics:**

Distal radioulnar joint dislocation, DRUJ, DRUJ dislocation, elbow dislocation, orthopedics, ortho, upper extremity, wrist injury, elbow injury, closed reduction.

**Figure:**
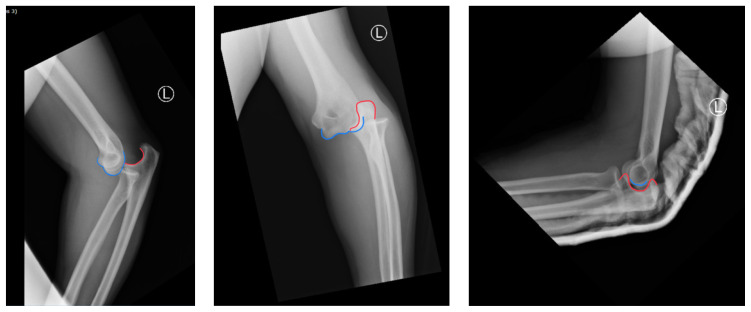

**Figure:**
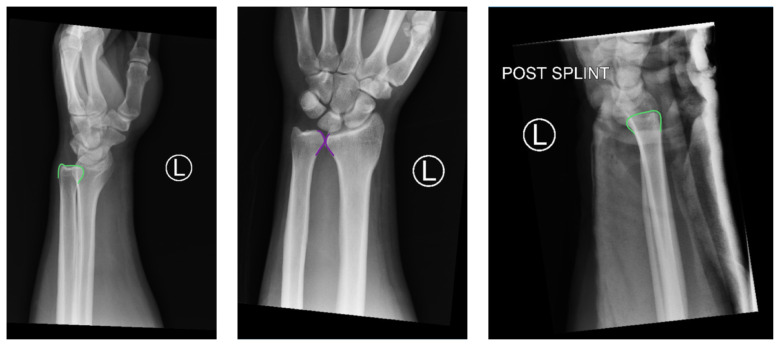

## Introduction

The distal radioulnar joint (DRUJ) plays a critical role in pronation and supination of the forearm and can be injured with traumatic axial loading of the wrist.^1^ This case report describes a patient who had a fall onto an outstretched hand (FOOSH) resulting in both a posterolateral elbow dislocation and a dorsal DRUJ dislocation. Because elbow dislocations are readily identified on radiographs, but DRUJ injuries can be much more subtle and are often missed on initial assessment,^2^ this case report focuses on the DRUJ dislocation.

## Presenting concerns and clinical findings*:*

A 36-year-old male presented to the emergency department with left upper extremity (LUE) pain after falling from ground level onto his outstretched arm. His pain was 10/10 throughout the left upper extremity and hard for him to localize. He denied head injury or loss of consciousness and had no other complaints. There was obvious swelling to the left elbow with tenderness and decreased range of motion (ROM) of the left elbow and wrist due to pain. He had no pain with active or passive range of motion of his left shoulder or left fingers. The skin and neurovascular exams of the extremity were intact. He had no other signs of trauma or concerning physical exam findings.

## Significant findings

Radiographs of the left elbow and wrist were obtained. Left elbow radiographs showed simple posterolateral dislocation of the olecranon (red) without fracture of the olecranon (red) or trochlea (blue). Left wrist lateral radiographs demonstrated DRUJ dislocation with dorsal displacement of the distal ulna (green) without fracture or widening of the radioulnar joint (purple). Post-reduction radiographs demonstrated appropriate alignment of the elbow with the trochlea seated in the olecranon and improved alignment of the DRUJ.

## Patient course

Orthopedics was consulted in the ER and performed a closed reduction of the patient’s left elbow and wrist after the patient received intravenous pain meds. The patient’s LUE was then immobilized in a posterior long arm splint with elbow flexed and forearm supinated to prevent recurrent dislocation of the left elbow and DRUJ. His pain significantly improved after reduction and splinting. The patient was given non-weight bearing instructions for his LUE and scheduled to follow up in one week in the orthopedics clinic. After a week, repeat films showed proper alignment of the DRUJ and elbow. Repeat physical exam showed only slight limitation of ROM from pain at both joints and stability of the DRUJ. His splint was removed and the patient was sent home with ROM exercises.

## Discussion

The distal radioulnar joint (DRUJ) is the articulation between the distal radius and the ulna. In conjunction with the proximal radioulnar joint, forearm bones, and interosseous membrane, the DRUJ allows for pronation, supination, and load transmission across the wrist. It also consists of the radioulnar ligaments and the primary soft tissue stabilizer of the joint, the triangular fibrocartilage complex (TFCC). Injuries of the DRUJ are not uncommon.^1^ They may be isolated to the soft tissue alone, or more commonly, associated with fractures of the radius or ulna. Patients with DRUJ injuries present with ulnar wrist pain and may have instability of the joint or limitation in pronation or supination on physical exam.^3^

Traumatic injuries, such as a fall onto an outstretched hand (FOOSH), with axial loading of an extended and pronated wrist cause dorsal displacement of the distal ulna in relation to the radius, resulting in the more common dorsal DRUJ dislocation. Emergency medicine (EM) physicians should complete a full neurovascular exam and obtain radiographs on all patients with FOOSH injuries. On true lateral radiograph films, DRUJ dislocation is established by the dorsal displacement of the ulna of approximately 6 mm or more from the radius (normal is approximately 2 mm). On anteroposterior films, there may also be a widened joint space between the distal radius and ulna.^4^ Volar dislocations also occur, although less frequently. Due to a lack of obvious deformity on physical exam as well as unfamiliarity with the finding on wrist films, up to 50% of DRUJ dislocations are missed on initial assessment which means that EM physicians should have a high index of suspicion.^2^

DRUJ dislocations are described as simple if they are easily reduced or complex if soft tissue entrapment prevents reduction. Reduction of a DRUJ dislocation can be performed by EM physicians and does not always require orthopedic consultation. Reduction of simple dorsal dislocations is achieved by supination at the wrist with volar directed pressure at the distal ulna. Volar dislocations are reduced with pronation.^5^ Most DRUJ injuries are initially treated with conservative measures such as NSAIDs and immobilization with splinting.^1^ Dorsal dislocations should be splinted with the wrist immobilized in supination and volar dislocations with the wrist splinted in pronation.^1^ Patients should be referred for urgent outpatient orthopedic follow up as persistent instability after several weeks of immobilization, severe instability upon initial presentation, or complex dislocation may require operative management for pinning and radioulnar ligament repair.^6^ Undiagnosed or improperly managed DRUJ dislocations cause chronic instability, clicking, weakness or pain of the joint. Chronic instability that fails conservative management of reduction and immobilization may require more complicated ligamentous reconstruction by a hand surgeon to avoid these sequelae.^1^

While DRUJ dislocations associated with posterior elbow dislocations are not well documented in the literature, this case serves as an important reminder for EM physicians to consider alignment of the DRUJ on lateral wrist radiographs. It also highlights the importance of evaluating the joint above and below the primary injury in orthopedic cases. Concomitant wrist injuries should always be considered when evaluating or treating injuries to the elbow or proximal forearm. Early identification and management of these injuries can improve functional outcome by preventing chronic weakness or arthritis. Awareness of this commonly missed injury is important to ensure the correct diagnosis, consultation, treatment and follow up of affected patients.

## Supplementary Information
























