# Supplementary figures and images for: Case Report of Distal Radioulnar Joint and Posterior Elbow Dislocation
Source: J Educ Teach Emerg Med. 2020 Oct 15;5(4):V12–4. doi: 10.21980/J89S6K (PMC10332524; doi:10.21980/J89S6K)

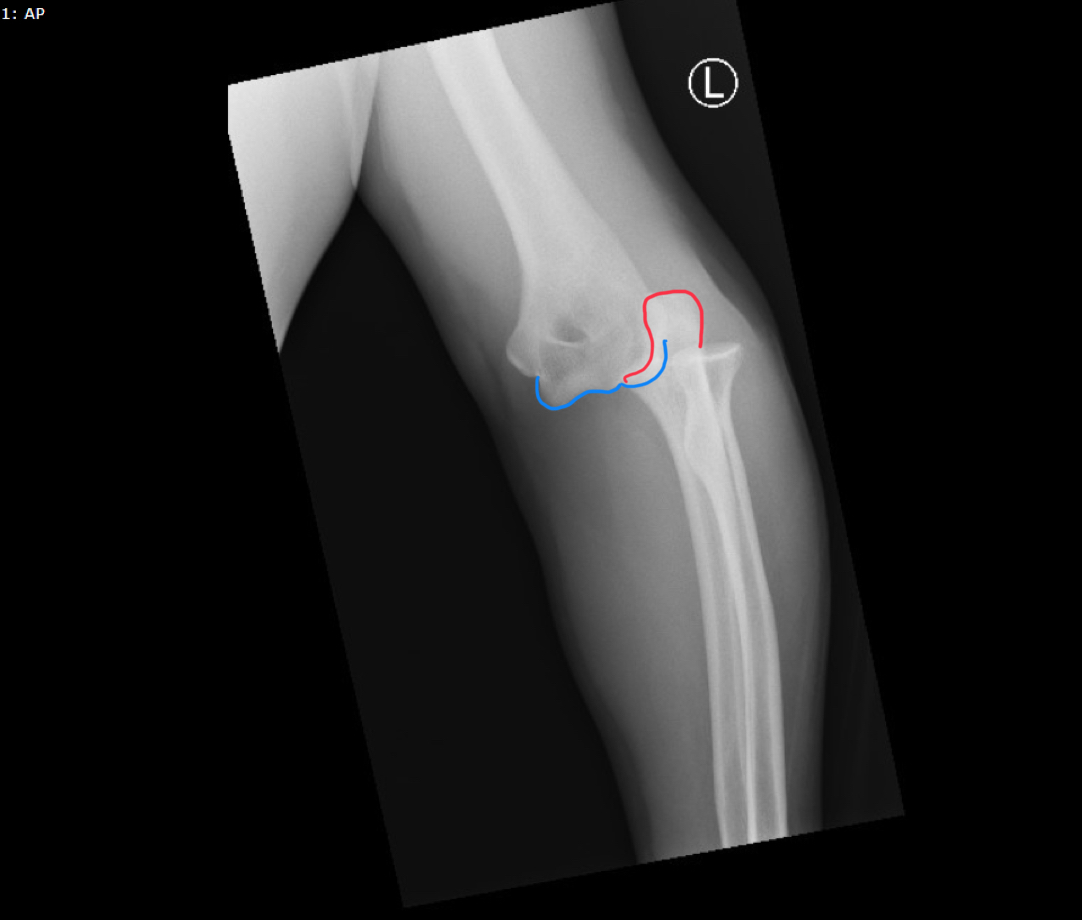

Supplement: Supplementary file 1 [file jetem-5-4-v12-supp1.jpeg]

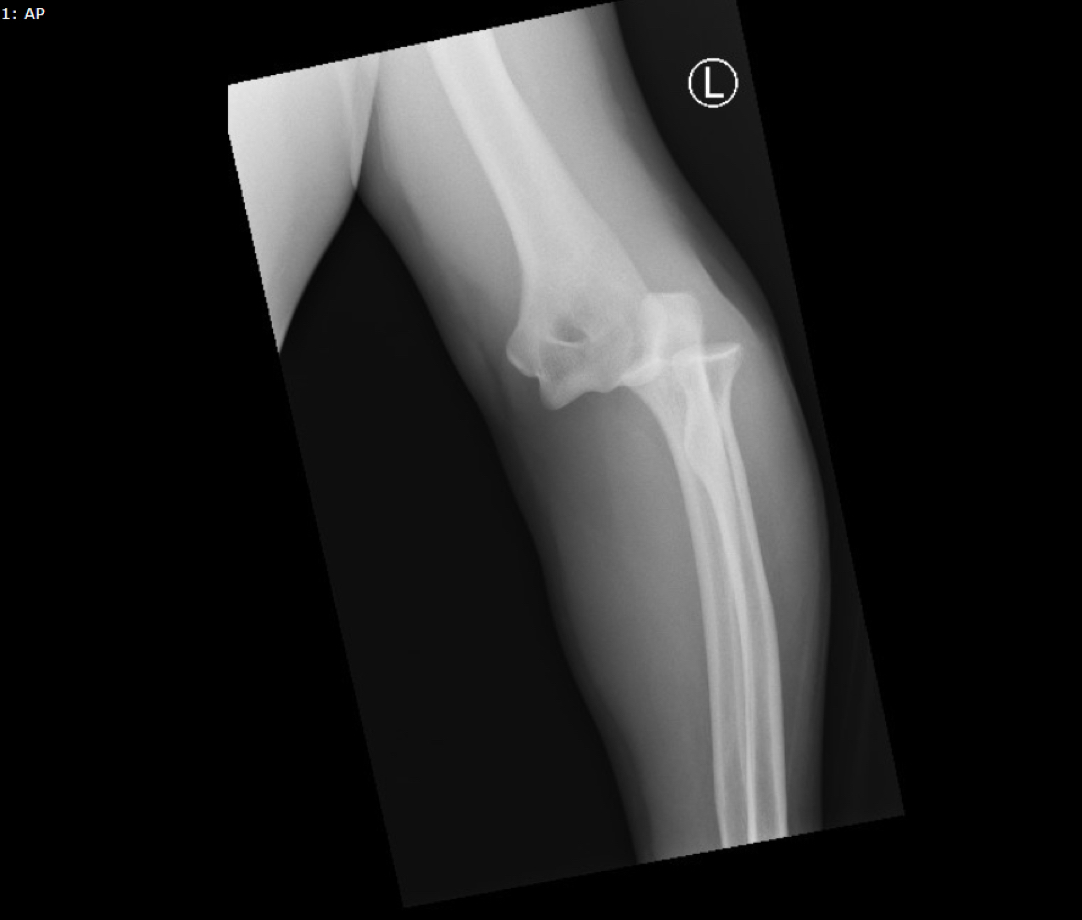

Supplement: Supplementary file 2 [file jetem-5-4-v12-supp2.jpeg]

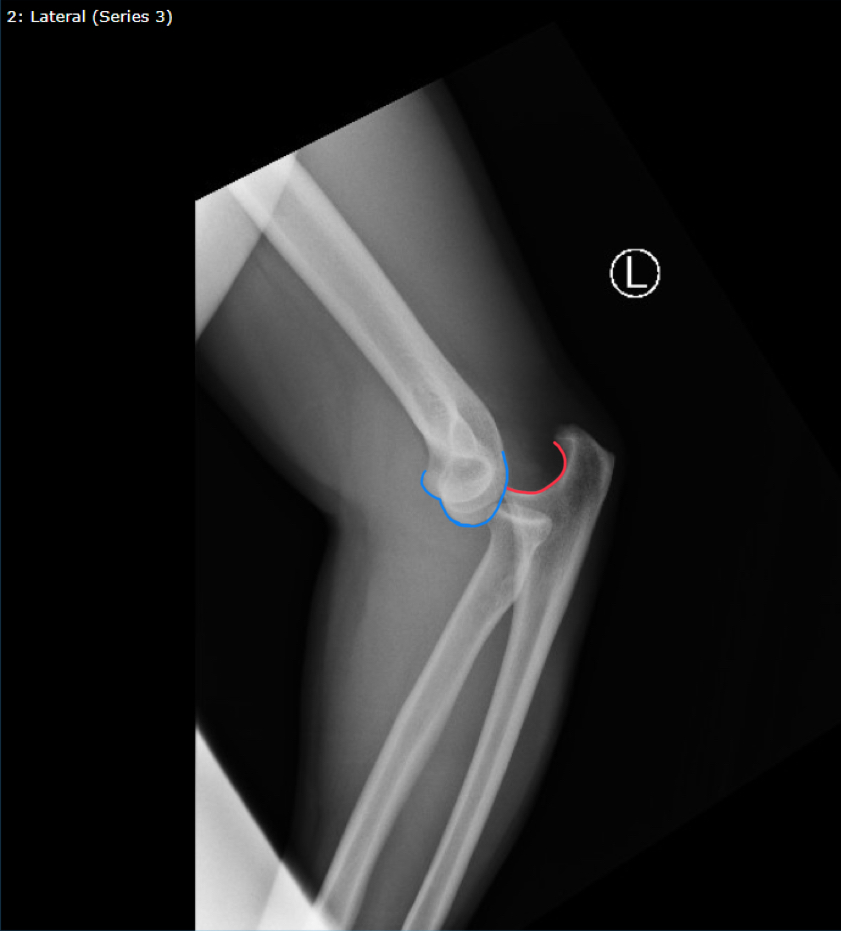

Supplement: Supplementary file 3 [file jetem-5-4-v12-supp3.jpeg]

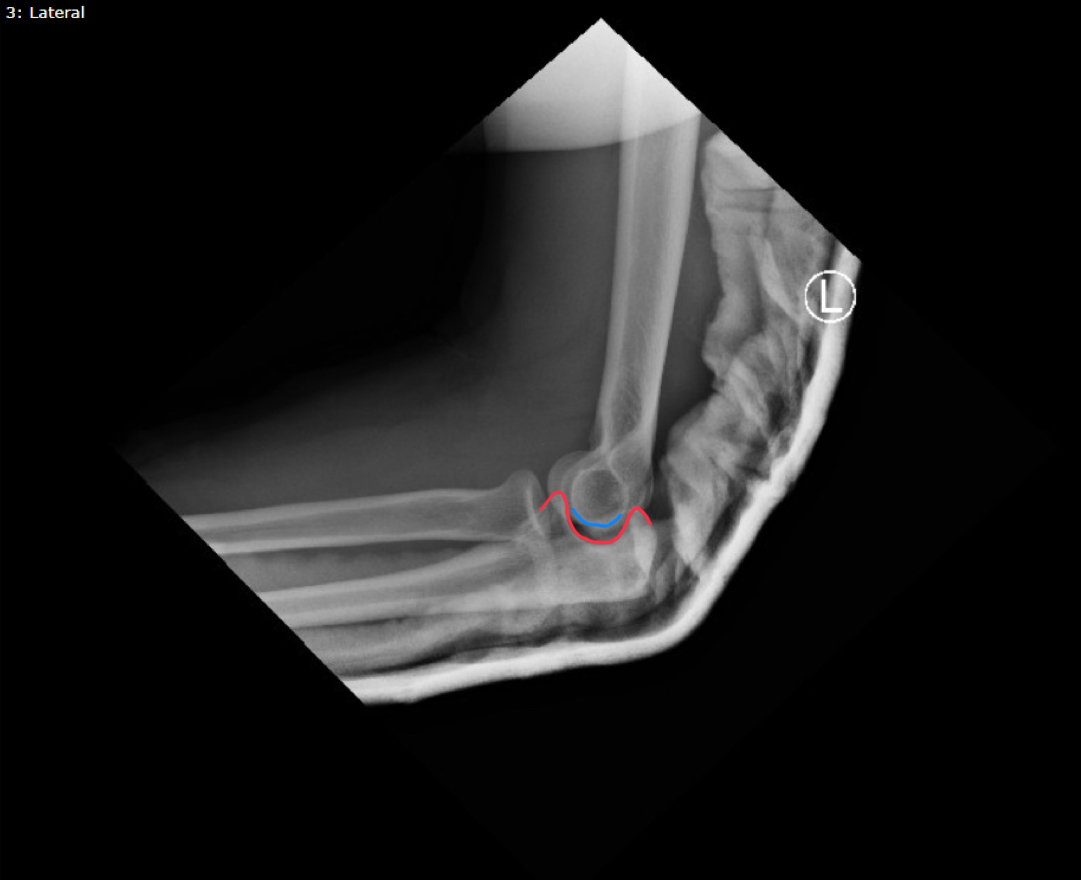

Supplement: Supplementary file 4 [file jetem-5-4-v12-supp4.jpeg]

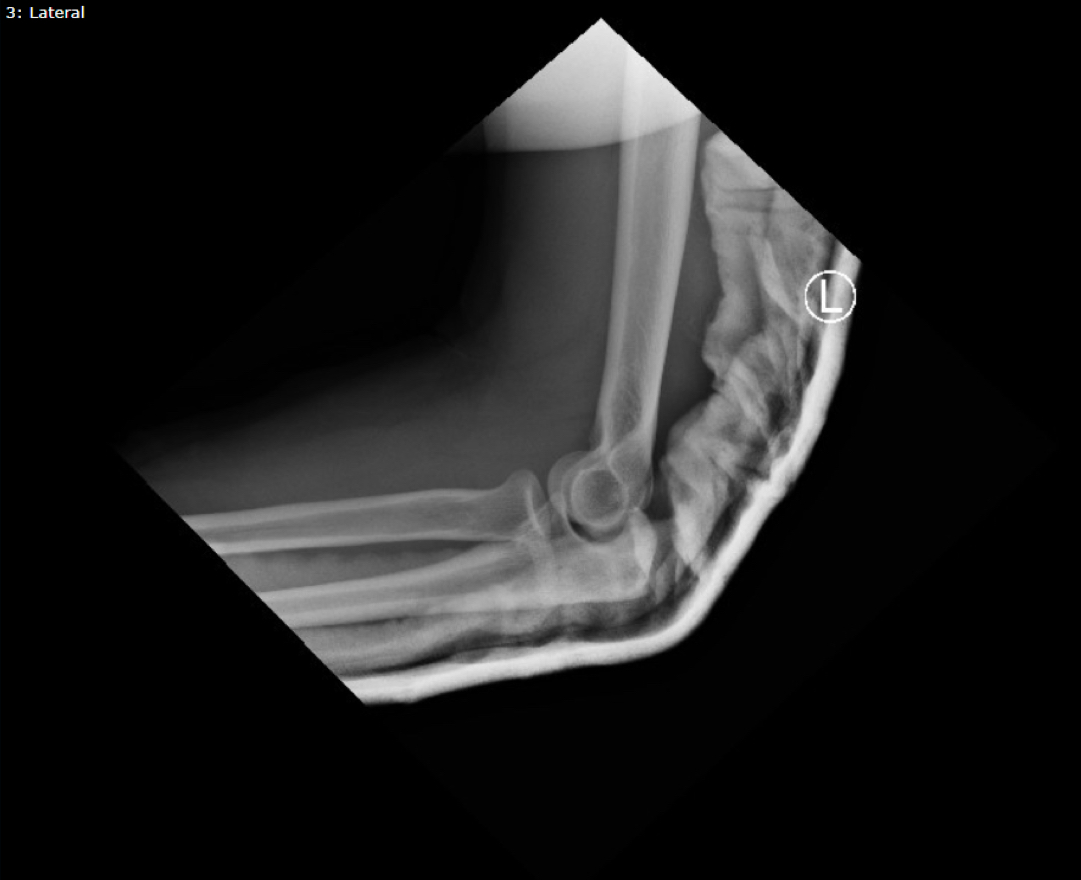

Supplement: Supplementary file 5 [file jetem-5-4-v12-supp5.jpeg]

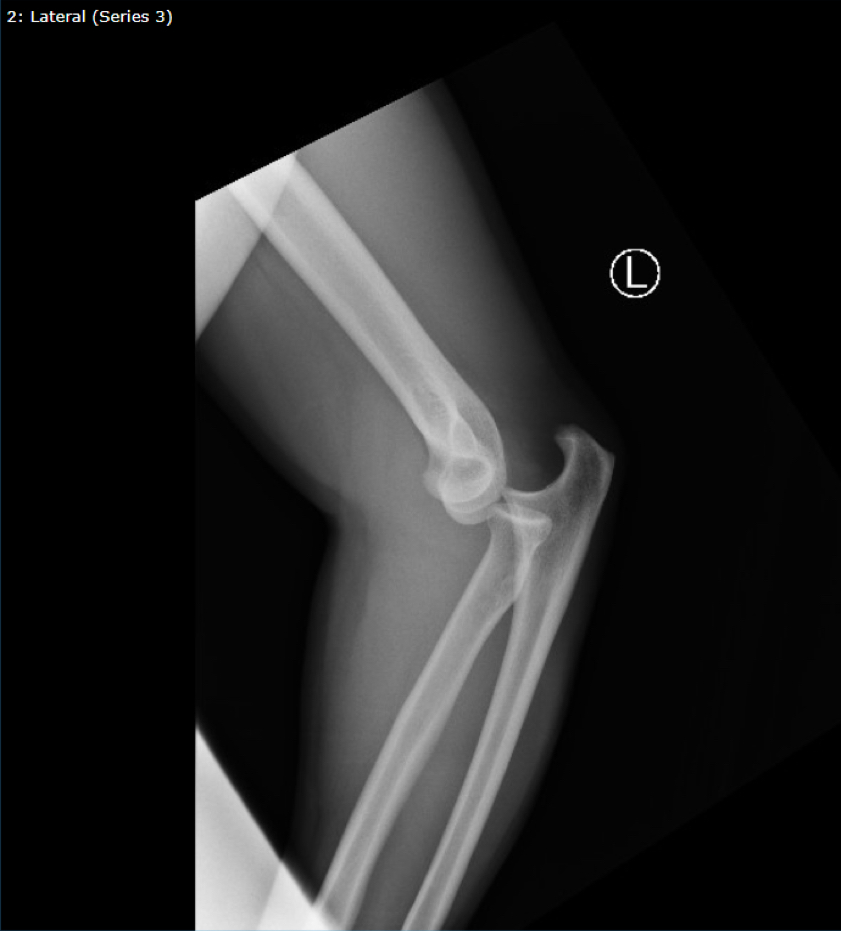

Supplement: Supplementary file 6 [file jetem-5-4-v12-supp6.jpeg]

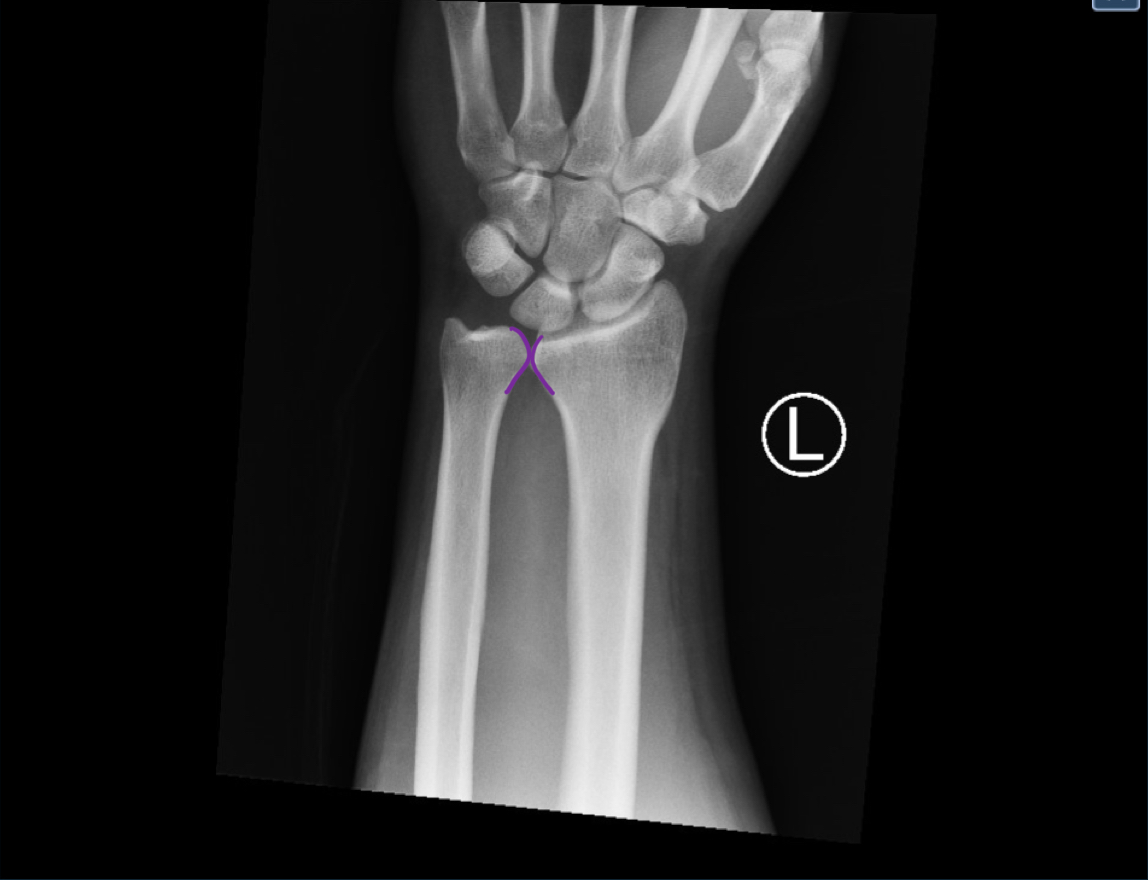

Supplement: Supplementary file 7 [file jetem-5-4-v12-supp7.jpeg]

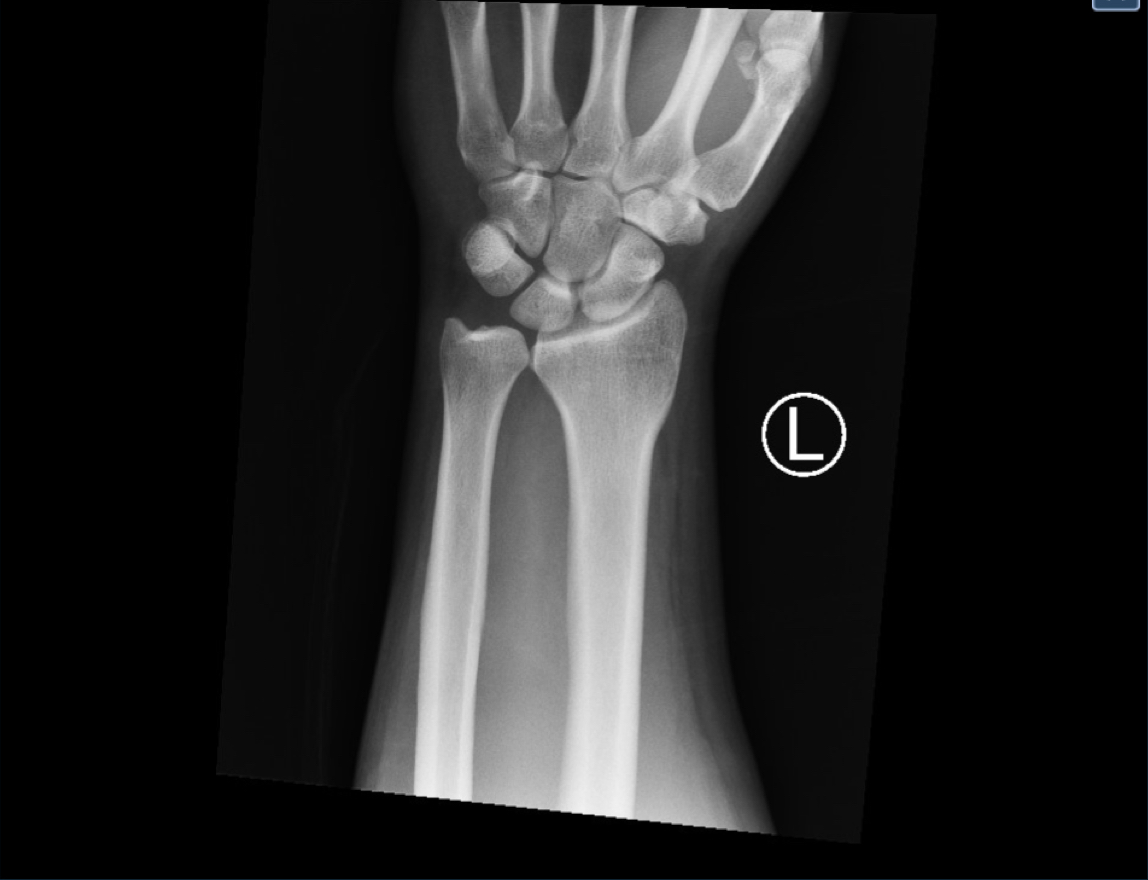

Supplement: Supplementary file 8 [file jetem-5-4-v12-supp8.jpeg]

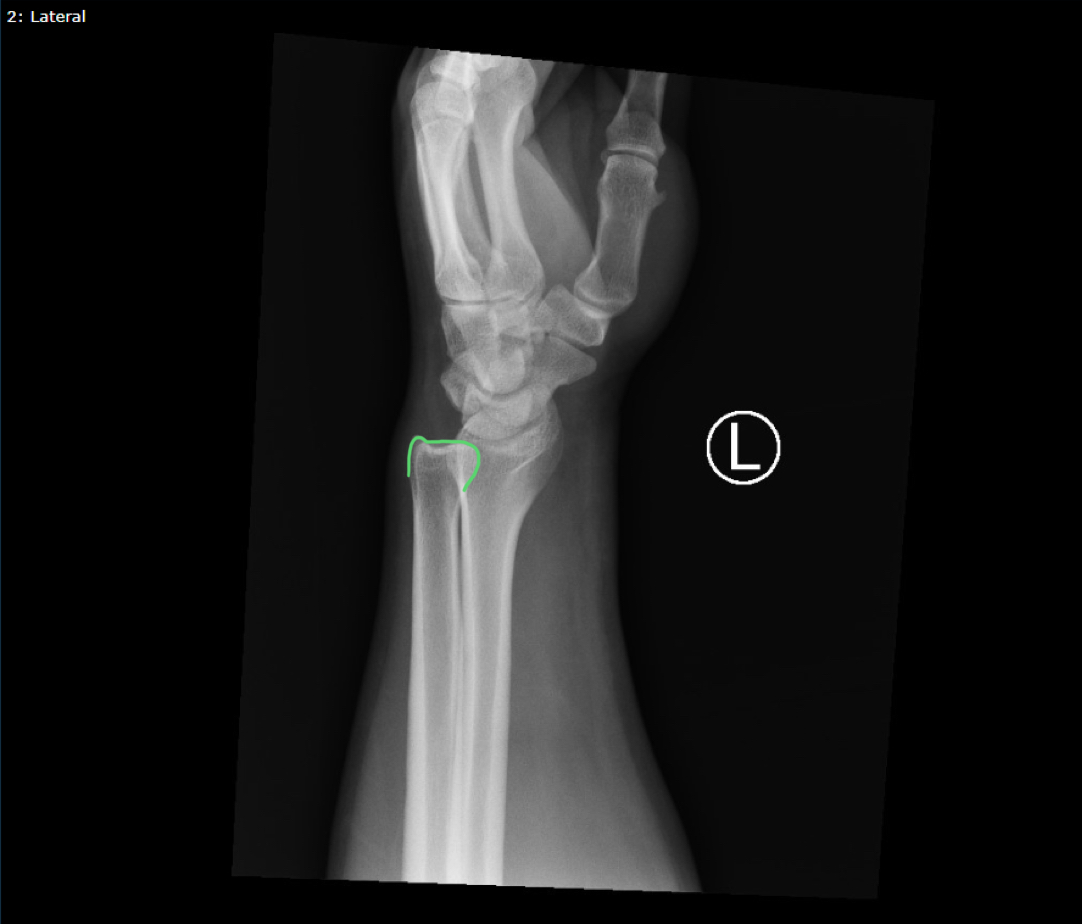

Supplement: Supplementary file 9 [file jetem-5-4-v12-supp9.jpeg]

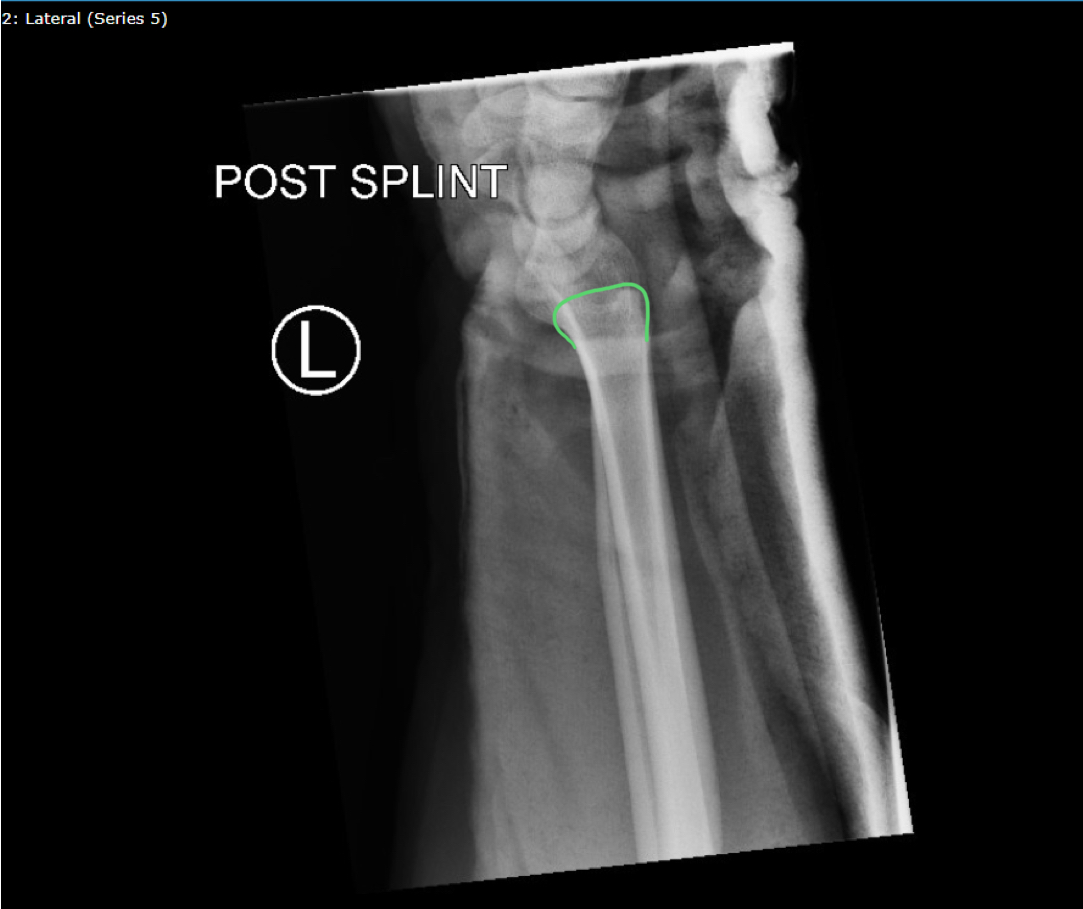

Supplement: Supplementary file 10 [file jetem-5-4-v12-supp10.jpeg]

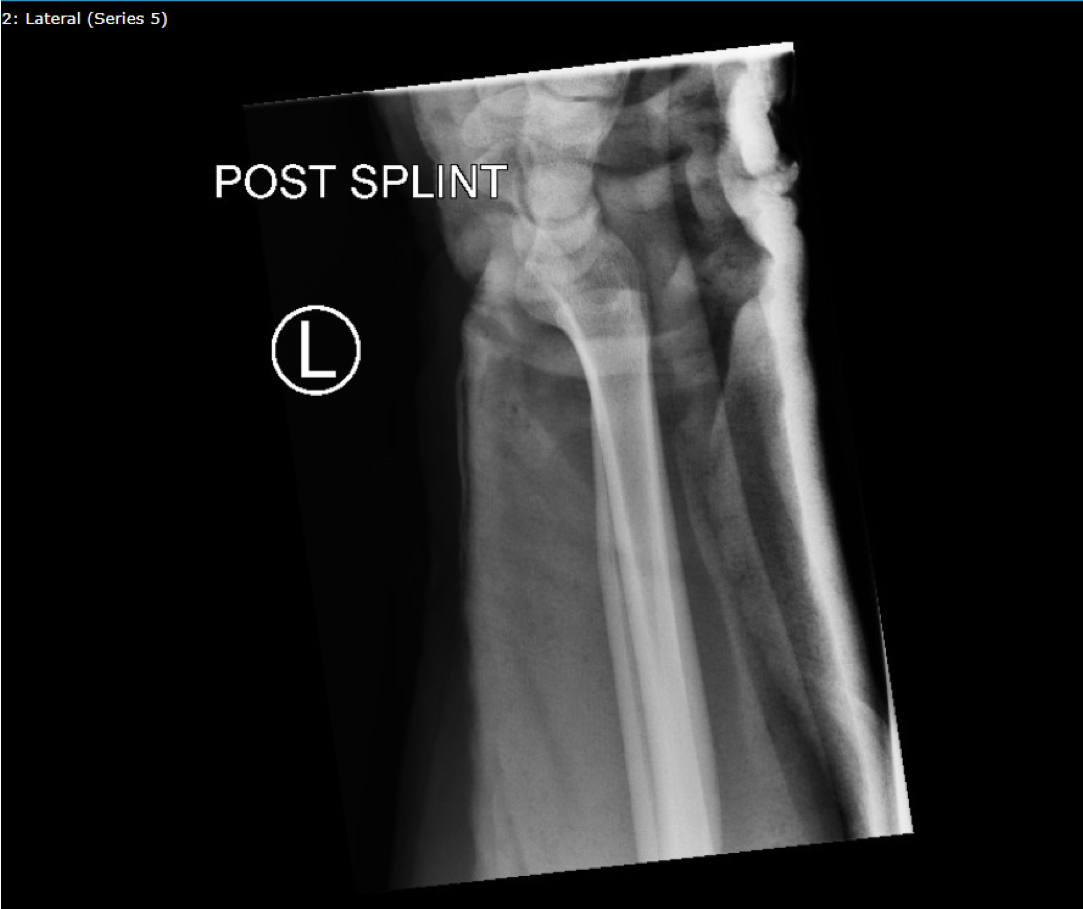

Supplement: Supplementary file 11 [file jetem-5-4-v12-supp11.jpeg]

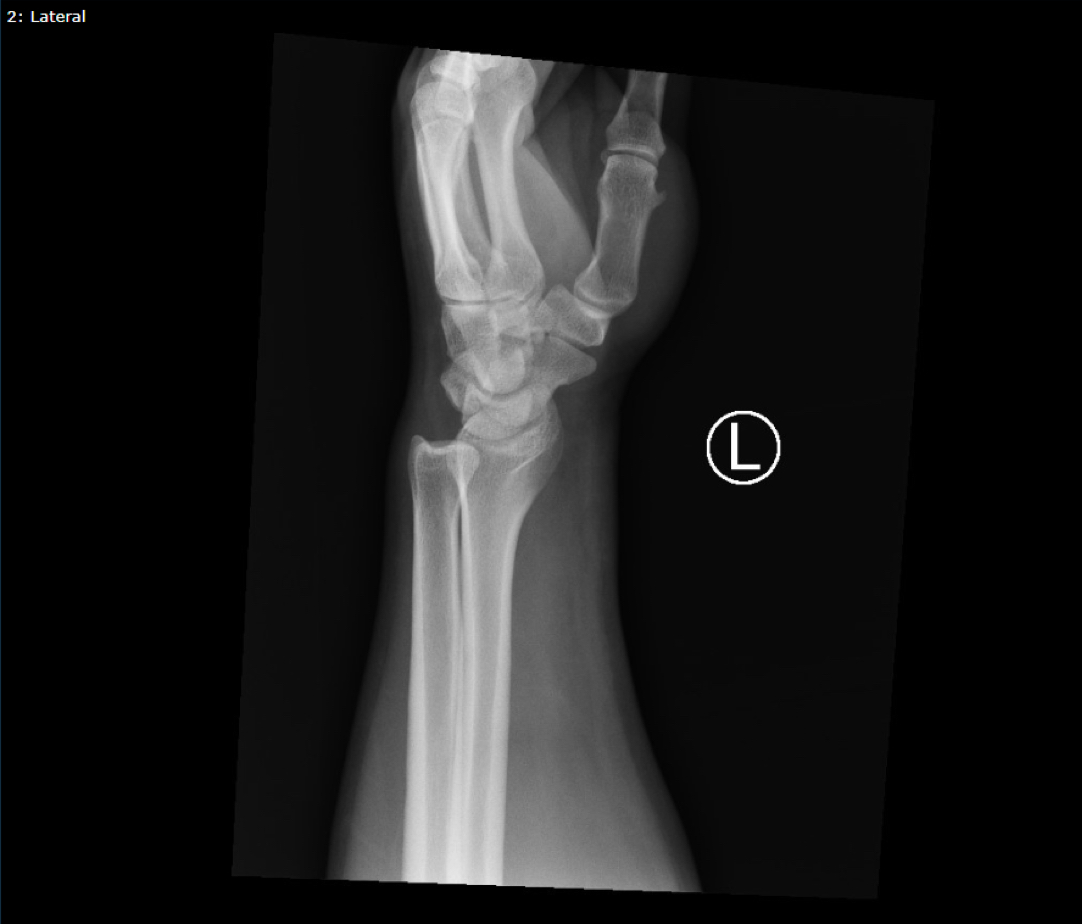

Supplement: Supplementary file 12 [file jetem-5-4-v12-supp12.jpeg]
